# Metabolic Remodeling Impacts the Epigenetic Landscape of Dental Mesenchymal Stem Cells

**DOI:** 10.1155/2022/3490433

**Published:** 2022-04-05

**Authors:** Haiyun Luo, Yachuan Zhou, Wenjing Liu

**Affiliations:** ^1^Stomatological Hospital, Southern Medical University, Guangzhou 510280, China; ^2^State Key Laboratory of Oral Diseases & National Clinical Research Center for Oral Diseases & Department of Cariology and Endodontics, West China Hospital of Stomatology, Sichuan University, Chengdu 610041, China

## Abstract

Epigenetic regulation can dynamically adjust the gene expression program of cell fate decision according to the cellular microenvironment. Emerging studies have shown that metabolic activities provide fundamental components for epigenetic modifications and these metabolic-sensitive epigenetic events dramatically impact the cellular function of stem cells. Dental mesenchymal stem cells are promising adult stem cell resource for *in situ* injury repair and tissue engineering. In this review, we discuss the impact of metabolic fluctuations on epigenetic modifications in the oral and maxillofacial regions. The principles of the metabolic link to epigenetic modifications and the interaction between metabolite substrates and canonical epigenetic events in dental mesenchymal stem cells are summarized. The coordination between metabolic pathways and epigenetic events plays an important role in cellular progresses including differentiation, inflammatory responses, and aging. The metabolic-epigenetic network is critical for expanding our current understanding of tissue homeostasis and cell fate decision and for guiding potential therapeutic approaches in dental regeneration and infectious diseases.

## 1. Introduction

Adult mesenchymal stem cells (MSCs) residing in various tissues still have the capacity to undergo differentiation and self-renewal. Most MSCs stay quiescent in local tissues and can differentiate into certain cell types upon specific signaling, which plays a critical role in tissue homeostasis maintenance and regenerative need preservation [1]. Multiple adult stem cell types with diverse biological properties have been reported in many tissues and organs, including the bone marrow, blood, muscle, skin, and teeth. Stem cells derived from dental tissue include dental pulp stem cells (DPSCs), periodontal ligament stem cells (PDLSCs), stem cells from the apical papilla, and stem cells from human exfoliated deciduous teeth. Dental mesenchymal stem cells can undergo odontogenic, osteogenic, neurogenic, and chondrogenic differentiation, which contribute to injury repair of oral and maxillofacial tissues [2]. As a promising cell resource in tissue engineering, dental mesenchymal stem cells possess superior pluripotency and regenerative potential compared with other MSCs and can be easily obtained from extracted teeth [3, 4]. Clarifying the cellular mechanism in the cell fate decision of dental mesenchymal stem cells will greatly benefit therapeutic approaches in local injury repair and stem cell-based tissue engineering.

Cell fate decision is driven by a highly coordinated transcriptomic program and epigenetic events. There are multiple regulators and crucial features that contribute to the mechanism by which cells make choice and determination. During cell fate decision, a cell transition state is an intermediate stage with mixed identity, which could be kind of unstable and reversible [5]. Emerging evidence has suggested that dynamic epigenetic modifications play an important role in the cellular function of stem cells, including differentiation, self-renewal, and apoptosis [6]. Genomic DNA can be expressed differently due to alterations in chromatin structure constructed by nucleosome core particles. For each nucleosome, 147 base pairs of DNA were wrapped around a histone octamer (including histone proteins H2A, H2B, H3, and H4). The methylation modification in the cytosine-guanine (CpG) islands of the DNA promoter regions leads to the inhibition of transcription [7]. Histones can be dynamically modified with various chemical groups and form methylation, acetylation, ubiquitylation, or other modifications [8, 9]. Histone modifications play a critical role in regulating the chromatin organization and accessibility of DNA to proteins [10]. Histone acetylation mainly occurs on lysine residues of histones that would weaken the charge attraction between histones and DNA and loosen the chromatin architecture, thereby facilitating gene transcription [11, 12]. Histone methylation also alters chromatin structure and has diverse effects on transcription control depending on the specific residues and number of methyl groups. For example, methylation of H3K9 and H3K27 is known as a signal of chromatin compaction and silencing [13], while trimethylation of H3K4 is primarily associated with promotor and transcription activation [14, 15]. After transcription, modified reactions can also occur on the RNA. As the most prevalent RNA modification, N^6^-methyladenosine (m^6^A) is an abundant methylation on adenosine residues that modulates every step of RNA metabolism, including processing, degradation, and translation [16]. Reversible modifications in DNA, histones, and RNA are involved in the temporal and spatial control of gene programs without alterations in nucleotide sequences.

Epigenetic regulation of the coordinated transcriptomic profile at the posttranscriptional level is required for cellular reprogramming, tissue homeostasis, and regeneration in response to environmental cues and metabolic signaling [17, 18]. For a long time, metabolic states have been recognized as consequences defined by cellular inputs and demands in specific stages. Cellular function and tissue homeostasis are supported by the cooperation of several metabolic pathways, such as glycolysis, mitochondrial oxidative metabolism, and fatty acid catabolism [19, 20]. Metabolic remodeling during cell fate transition is accompanied by fluctuations of various metabolites [21, 22]. Beyond energy production and biomass synthesis, metabolic fluctuations are now found to be essential cues in the regulation of self-renewal and differentiation, with technological advancements. The dynamic metabolic pattern is coordinated with the stage-specific requirements and nutrition availability to different cells in the microenvironment [23]. Metabolic intermediates such as methyl groups, *α*-ketoglutarate (*α*-KG), and acetyl coenzyme A (acetyl-CoA) are key substrates and cofactors for enzyme activities in epigenetic events that act as the bridge between the intracellular microenvironment and cell biology [23, 24]. Great strides have been made in the transcriptional pattern and epigenetic regulatory network during cell fate transition and decision [25]. Recent studies have addressed the metabolic cues in epigenetic regulation that overcome the knowledge gaps in understanding cell fate decisions [26–28]. The metabolic-epigenetic network could shed light on the cellular mechanism of dental mesenchymal stem cell fate decision.

In the present study, we review the conceptual foundation of the link from metabolism to epigenomics and the regulatory effect of epigenetic modifications on dental mesenchymal stem cells. Furthermore, we summarize the current knowledge of metabolic pathways and explore the potential role of metabolism as an epigenetic regulator in the oral and maxillofacial regions.

## 2. The Link from Metabolism to Epigenomics in Dental Mesenchymal Stem Cells

In addition to the well-known modifications of methylation, acetylation, and ubiquitination, there are many other less-known modifications, such as acylation and glycosylation, which comprise more than one hundred modifications in the epigenetic region [23]. Although the establishment of different modifications is complex and specific, there are some basic principles in this process that will help us understand the interaction between metabolic pathways and epigenetic events. Epigenetic reactions rely on the availability of corresponding chemical groups, cofactors, or antagonists derived from cellular metabolism [23, 29]. Chromatin-modifying enzymes such as methyltransferase and acetyltransferase are capable of utilizing metabolite substrates to install chemical marks, which can also be removed by specific enzymes. Epigenetic dynamics depend on the balance between the thermodynamic (*K*_d_ value) and kinetics (*K*_m_ value) of enzymes and the intracellular concentrations of corresponding metabolic substrates [29, 30]. In this way, metabolism plays an essential regulatory role in enzyme activity and subsequently defines the modifications in chromatin and RNA. Given the differences in the kinetic and thermodynamic properties of epigenetic enzymes and the availability of metabolite intermediates, chemical reactions display diverse sensitivities with metabolic states. Epigenetic modifications such as methylation and acetylation are canonical reactions in which enzyme activities are sensitive to the intracellular concentrations of metabolite substrates. Some other modifications seem to be less sensitive and not responsive to metabolic fluctuations. For example, ubiquitination reactions require ATP metabolites to form modifications, while the intracellular level of ATP is far more than the requirement [31]. Moreover, there are other mechanisms that link metabolic pathways to epigenetic events, permitting the complexity and plasticity of the metabolism-epigenetic network. Epigenetic modifications can be established without enzyme activity in certain situations, even for typical modifications such as methylation and acetylation [32]. Cell fate decision can be induced by altering the epigenetic sets of critical regulatory factors [10]. Global chromatin modification preferentially impacts the transcriptional dynamics of specific genes that are highly associated with cellular identity and function [33–35]. Metabolic remodeling reshapes the epigenetic landscape by inducing new epigenetic states or changing specific modification reactions. These metabolic-related epigenetic alterations further impact the transcriptional program and participate in the cell fate decision [27, 28]. Current studies regarding epigenetic regulation in the oral and maxillofacial regions have mainly focused on the functional effects of epigenetic modifying enzymes, while the regulatory role of metabolism remains elusive. In the following sections, we discuss the interaction of metabolic fluctuations and context-dependent epigenetic events during the cell fate decision of dental mesenchymal stem cells.

### 2.1. The Impact of Metabolism on Methylation

Methylation conducted by methyltransferases can occur on DNA, histones, and RNA. DNA and histone methylation in chromatin are characterized by methyl group transfer conducted by DNA methyltransferases (DNMTs) and histone methyltransferases (HMTs) [7, 9]. RNA m^6^A methylation is catalyzed by the methyltransferase-like 3 (METTL3)/METTL14 complex [36]. DNA methylation profiles in whole genomes of odontogenic cell populations show that the differences in DNA methylation are correlated with the osteogenic capacity of dental mesenchymal stem cells [37]. DNA methylation also participates in the immune and inflammatory responses of dental mesenchymal stem cells by regulating inflammatory cytokine secretion and signaling pathways [38, 39]. For histone methylation, the trimethylation of histone 3 lysine 27 (H3K27me3) regulates DPSC differentiation by suppressing the expression of odontogenic-related genes and activation of the Wnt/*β*-catenin pathway [40]. Other histone methylations such as H3K9me2, H3K9me3, and H3K4me3 also participate in the differentiation processes of DPSCs and PDLSCs [41–43]. Additionally, high levels of H3K27me3 and H3K4me3 inhibit cytokine secretion and corresponding pathway activation during the inflammatory response [44, 45]. METTL3-mediated RNA m^6^A methylation modulates tooth root development [46] and regulates DPSC apoptosis via cell cycle progression [47]. METTL3 is also involved in the inflammatory response of DPSCs by modulating the alternative splicing of RNA [48].

**Figure 1:**
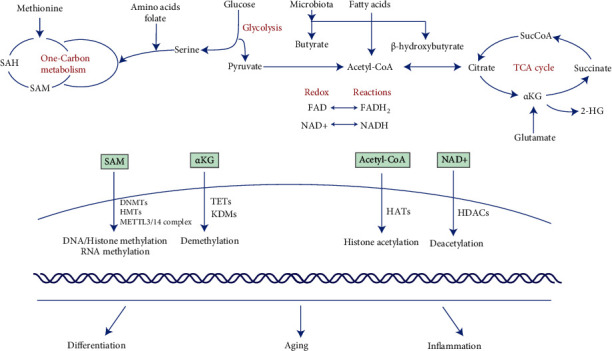
Amino acids, glucose, and fatty acids are utilized by metabolic pathways including one carbon metabolism, glycolysis, TCA cycle, and fatty acid catabolism. Metabolic intermediates generated from cellular metabolic pathways are substrates, cofactors, or antagonists for enzyme activity in epigenetic events including methylation, demethylation, acetylation, and deacetylation. These metabolic-sensitive epigenetic events are involved in the transcriptional program alteration and drive the dental mesenchymal stem cells into differentiation, aging, or inflammatory responses. SAM: S-adenosylmethionine; SAH: S-adenosylhomocysteine; acetyl-CoA: acetyl coenzyme A; SucCoA: succinyl-CoA; 2-HG: 2-hydroxyglutarate; *α*KG: *α*-ketoglutarate; TCA cycle: tricarboxylic acid cycle; NAD: nicotinamide adenine dinucleotide; FAD: flavin adenine dinucleotide; DNMTs: DNA methyltransferases; HMTs: histone methyltransferases; METTL3: methyltransferase-like 3; TETs: ten-eleven translocations; KDMs: histone lysine demethylases; HATs: histone acetyltransferases; HDACs: histone deacetylases.

The methyl donor S-adenosylmethionine (SAM) in these methylation reactions is primarily provided by one-carbon metabolism, which generates one-carbon units (methyl groups) for biosynthetic processes [49, 50]. One-carbon metabolism is a network of interconnected metabolic pathways, including the methionine cycle, folate cycle, and transsulfuration pathway [50]. SAM is an important metabolite substrate catalyzed by methionine adenosyltransferase in methionine metabolism. Other amino acids, such as threonine, serine, choline, and glycine, in the folate cycle can also interact with methionine metabolism and support SAM generation (Figure 1). Then, DNMTs, HMTs, and RNA methyltransferase complex can convert the SAM to S-adenosylhomocysteine (SAH), which supplies the methyl groups for DNA/histone and RNA methylation [51, 52]. The ratio of SAM/SAH is recognized as the intracellular methylated potential that impacts enzymatic activities.

The methyl donors and intermediates generated from one-carbon metabolism drive epigenetic reprogramming for the cell fate decision [49]. Accumulating studies have suggested a regulatory effect of one-carbon metabolism on global DNA methylation levels, and nutrient deficiency can lead to DNA hypomethylation [53]. Methionine and threonine support DNA and histone methylation in embryonic stem cells, which are essential for self-renewal and pluripotency maintenance [54]. The reduction in SAM accumulation due to threonine depletion leads to cell differentiation by reducing H3K4me3 levels [55]. Serine metabolism also regulates neural stem cell differentiation by modulating the methylation state of H3K4 [56]. In LPS-induced macrophages, activation of one-carbon metabolism promotes SAM generation and supports the trimethylation level of H3K36 on interleukin-1*β* [57].

In dental mesenchymal stem cells, amino acid levels change in response to physiological stimulation, which leads to the alteration of metabolite substrates in one-carbon metabolism [58, 59]. Yang et al. demonstrated that the differences in one-carbon metabolism of young DPSCs and aging DPSCs contribute to the alteration of regenerative capacity [59]. Serine, glycine, and threonine are significantly suppressed in aging DPSCs which restrict the SAM supply, resulting in low DNA methylation of the senescence marker p16 [59].

### 2.2. The Impact of Metabolism on Demethylation

Methylation marks can be reversibly turned over by demethylases in coordination with the metabolic state. Demethylases are capable of removing the methyl groups from DNA, histones, and RNA in a context-dependent manner. Active removal of DNA methylation can be catalyzed by the ten-eleven translocation proteins (TETs) including TET1, TET2, and TET3, which are *α*-KG-dependent dioxygenases [23, 60]. TET1 is upregulated during DPSC mineralization and facilitates odontogenic differentiation [61]. TET1 and TET2 are also involved in modulating the immunomodulatory response of PDLSCs [62]. For histone demethylation, Jumonji domain-containing demethylase 3 (JDJM3) and another demethylase, lysine demethylase 5A (KDM5A), regulate the odontogenic differentiation by inhibiting the trimethylation levels of H3K27 and H3K4 on odontogenic-related genes, respectively [63–65].

The catalytic activities of some critical demethylases such as TETs and JMJDs depend on the metabolite substrate, *α*-KG and oxygen. *α*-KG is an important intermetabolite generated from the tricarboxylic acid cycle (TCA cycle) of mitochondrial metabolism and deamination of glutamate. In the TCA cycle, *α*-KG is produced from isocitrate upon the activity of isocitrate dehydrogenase and then decarboxylated into isocitrate dehydrogenase by *α*-KG dehydrogenase [66]. *α*-KG in the TCA cycle can also be anaplerotically generated from glutamate by glutamate dehydrogenase or glutamine transaminase (Figure 1). The enzyme activities of the TETs and JMJD families also require other cofactors from metabolic pathways, such as oxygen, ascorbate, and ferrous iron [67, 68]. Meanwhile, analogues of *α*-KG, such as succinate, citrate, and fumarate and 2-hydroxyglutarate (2HG), can inhibit the catalysis of demethylases [67, 69]. Additionally, hypoxia also suppresses enzymatic activities, which leads to high levels of DNA and histone methylation by limiting the oxygen supply [70, 71]. The balance between *α*-KG and its antagonists in intracellular pools is essential for defining the functional activity of *α*-KG-dependent demethylases.

Glucose metabolism alteration leads to changes in energy production and metabolite generation, which are essential for the cell fate decision. The differentiation initiation of hematopoietic stem cells presents a shift from glycolysis toward mitochondrial oxidative metabolism [72]. Conversely, a marked switch to predominantly glycolytic activity in energy supply is required for neural stem cell differentiation [73]. The metabolic substrates generated from glycolysis and mitochondrial oxidative metabolism are essential for epigenetic regulation of gene expression [19]. In naïve embryonic stem cells, a high level of intracellular *α*-KG produced by both glucose and glutamine metabolism is critical for maintaining pluripotency [74]. *α*-KG accumulation promotes the demethylation of H3K27me3 and TET-related DNA demethylation, which in turn regulates target gene expression [74]. During adipocyte differentiation, *α*-KG suppresses the adipogenic gene expression and brown adipogenesis by reducing the H3K4me3 methylation level [75]. The switch between glycolysis and mitochondrial oxidative metabolism contributes to intracellular *α*-KG alterations that orchestrate the requirement of different stages in a cell type-dependent manner [76].

Glucose metabolism remodeling also occurs during the differentiation processes of dental mesenchymal stem cells. In the differentiation initiation (for 0-3 days), mitochondrial ATP production increases with glycolytic activity, which might result from the rapid upregulation of energy demand [77]. Another study suggests that a high level of phosphofructokinase-dependent aerobic glycolysis is essential for odontoblast differentiation after induction for 6 days [78]. Maity et al. examined the whole differentiation process of DPSCs (from 0 to 14 days) and found that lactate production increased while metabolic activity was reduced [43]. The shift from mitochondrial oxidative metabolism to the glycolytic pathway corresponded with the upregulation of H3K4me3 on autophagy-related genes [43]. Metabolic pathway alteration from mitochondrial oxidative metabolism to glycolysis also participates in inflammatory responses. PDLSCs underwent a switch from oxidative phosphorylation to glycolysis after *Porphyromonas gingivalis* infection, characterized by succinate accumulation, ROS upregulation, and HIF-1 pathway activation [79]. These metabolite alterations in the inflammatory state might enhance the global methylation level by suppressing the enzyme reactions conducted by demethylases.

### 2.3. The Impact of Metabolism on Histone Acetylation

Histone acetylation is another well-known epigenetic modification that is established by histone acetyltransferases (HATs) with acetyl donors. During osteo/odontogenic differentiation of PDLSCs and DPSCs, the acetylation of histone 3 lysine 9 (H3K9ac) and H3K27ac is significantly increased [80–82]. Histone lysine acetyltransferase 2A (KAT2A) enhances the osteogenic differentiation of PDLSCs by increasing the acetylation levels of H3K9 and H3K14 [83]. Histone acetyltransferase p300 conducts H3K9ac of odontogenic-related genes and modulates DPSC differentiation and proliferation [82]. p300-related H3K9 acetylation also impacts the inflammatory response of periodontitis [84].

The key acetyl group in histone acetylation is acetyl-CoA, which is generated in diverse metabolic pathways [85]. In glycolytic activity, acetyl-CoA is generated from pyruvate catalyzed by pyruvate dehydrogenase and subsequently forms citrate, which is acquired by the TCA cycle in mitochondrial metabolism (Figure 1). Citrate can also be lysed and reverted back to acetyl-CoA by ATP-citrate lyase [86]. Additionally, acetate produced from fatty lipid catabolism and ethanol metabolism can be converted into acetyl-CoA by acetyl-CoA synthetase, which is another important source of acetyl-CoA. The nutrition supply and enzyme activity cooperate to define the availability of acetyl-CoA for histone acetylation [30].

The glucose metabolism alteration in dental mesenchymal stem cell fate transition not only impacts the generation of *α*-KG and acetyl-CoA; high levels of glycolysis are essential for pluripotency maintenance of embryonic stem cells by supplying acetyl-CoA for histone acetylation. After differentiation initiation, acetyl-CoA is rapidly consumed in the TCA cycle, leading to a reduction in the acetylation of histones [87]. Meanwhile, aerobic glycolysis in active T cells sustains the acetyl-CoA supply and enhances the H3K9ac of interferon gamma, which is essential for cell differentiation [88]. ATP-citrate lyase promotes acetylation of histone H3 and skeletal muscle cell differentiation by regulating the availability of acetyl groups [89].

As mentioned before, the differentiation process of DPSCs is characterized by enhanced aerobic glycolysis levels, and this alteration fuels sufficient acetyl-CoA that can be utilized for histone acetylation [43, 90]. DPSCs with faster aging rates display a reduction in the ability to use glucose and fatty acids as energy resources [91]. The lack of acetyl-CoA might contribute to cell senescence and aging via histone acetylation inhibition, which needs to be verified. Epigenetic reactions are also involved in the translation of lipid metabolic cues into the developmental setting. Fatty acid metabolism is an important resource of acetyl groups that is essential for cell differentiation and tooth development [92]. Dyslipidemia induced by a high-fat diet leads to an elevation in dentin thickness and a reduction in pulp cavity diameter and dentin formation width [92, 93]. Short-chain fatty acids (SCFAs) derived from microbiota promote the murine incisor regeneration [94]. SCFA supplementation facilitates dental mesenchymal stem cell differentiation by inducing acetylation of histone 3 in BMP signaling, which might result from increasing intracellular acetyl-CoA concentration and HDAC inhibition [94].

### 2.4. The Impact of Metabolism on Histone Deacetylation

As DNA and histone methylation, the acetyl groups installed on histones can be removed by the functional activities of histone deacetylases (HDACs) in a context-dependent manner. HDACs can be classified into classes I, II, and IV, which are zinc-dependent enzymes, and class III (also referred to as sirtuins), which are nicotinamide adenine dinucleotide- (NAD-) dependent enzymes. HDACs have essential effects on the biological processes of odontoblast, osteoblast, and cementoblast differentiation [95–97]. Sirtuin1 (SIRT1) facilitates the cellular differentiation of DPSCs in tissue regeneration and participates in inflammatory response [97–99]. The histone acetylation level in the differentiation process of dental mesenchymal stem cells is a consequence of the balance between HATs and HDACs.

NAD+ serves as the key coenzyme in multiple metabolic pathways and the cosubstrate for three classes of enzymes, including the sirtuin family. NAD is generated from vitamin B3 in the forms of nicotinamide, nicotinic acid, nicotinamide riboside, and amino acid tryptophan via de novo synthesis [100]. As an electron receptor in redox reactions, NAD+ can be converted into its reduced form, NADH, for oxidative phosphorylation and ATP synthesis, which mainly occurs in mitochondrial pathways (Figure 1). The intracellular NAD+ concentration modulates the enzyme activity of sirtuins which further impacts transcription and chromatic stability by histone deacetylation. On the other hand, the metabolic substrates from fatty acid catabolism, such as butyrate and *β*-hydroxybutyrate, are capable of inhibiting the enzymatic function of class I and some class II HDACs. Butyrate is a kind of SCFA produced by microbiota, and *β*-hydroxybutyrate is generated in the process of fatty acid oxidation or ketogenesis [101, 102].

Metabolic fluctuations due to glucose metabolism remodeling and fatty acid oxidation modulate histone deacetylase function during cell fate decision [103]. Skeletal muscle stem cell activation is characterized by increased glycolysis metabolism and reduced intracellular NAD+ and NAD+-dependent SIRT1 activity, which induce histone acetylation and gene transcription [104]. Sirtuins are downregulated during the cellular process of senescence and aging, and increasing mitochondrial NAD+ levels can delay this process [105]. Sodium butyrate, which is widely used as an HDAC inhibitor, can induce neurogenic effects and suppress the inflammatory response via histone deacetylation [103, 106].

The cellular differentiation of dental mesenchymal stem cells is accompanied by a high level of glycolysis that results in a decreased NAD+/NADH ratio and reactive oxygen production [43, 107]. The histone acetyltransferase SIRT1 is slightly upregulated on day 1 and then decreases over time during DPSC differentiation [97, 108]. The expression pattern of sirtuins might be related to the decreased NAD+/NADH ratio, which remains to be clarified. Periodontal microbiota, such as *Porphyromonas* and *Fusobacterium*, can produce SCFAs, including butyrate and isobutyrate, which are known as resources for HDAC inhibitors [109, 110]. High concentrations of butyric acid derived from *Porphyromonas gingivalis* can suppress the expression levels of HDACs while inducing the acetylation of histone 3, which further promotes bone absorption in periodontitis [110, 111]. Butyric-induced histone acetylation is also involved in cell vitality, differentiation, and extracellular matrix remodeling of osteoblastic cells [109].

### 2.5. The Impact of Metabolism on Other Modifications

Beyond methylation and acetylation, there are also a growing number of modifications that add functional diversity to epigenetic regulation. Histone acylation is similar to histone acetylation in function and activity and relies on histone acetyltransferases to utilize acyl-CoA [112, 113]. Acyl-CoA molecules are metabolite substrates generated from short-chain acyl group-containing molecules, including SCFAs [114]. RNA acetylation relies on the same metabolite acetyl-CoA as histone acetylation to form N^4^-acetylcytidine and impacts RNA stability and translation efficiency [115]. Histone acylation and RNA acetylation exhibit some similarities with histone acetylation in enzyme activity or metabolite substrates; however, their biological function remains unknown in dental mesenchymal stem cells. Metabolite-derived modifications, such as histone homocysteinylation, histone monoaminylation, and histone ADP-ribosylation, also need to be explored in future studies [9, 116, 117].

## 3. Metabolism-Epigenetic Network in Dental Mesenchymal Stem Cells

The epigenetic landscape alteration driven by metabolic remodeling participates in a highly coordinated transcriptional program in a stage- and cell type-specific manner. On the other hand, cellular progress can also be partially reversed by the metabolism-epigenetic network. Dental mesenchymal stem cells retain plasticity and flexibility to adjust their cell biology toward the surrounding microenvironment. The present study discusses the potential impact of metabolic remodeling on epigenetic events during biological processes of dental mesenchymal stem cells, including differentiation, aging, and inflammation.

### 3.1. Differentiation

Metabolic remodeling in the cellular differentiation process orchestrates the emerging changes in energy supply and metabolite demand. According to previous studies, an increasing level of aerobic glycolysis was identified in DPSC differentiation based on indicators such as the oxygen consumption rate and extracellular acidification rate [43, 77, 78]. Metabolic remodeling could result in changes in the corresponding metabolites such as acetyl-CoA, *α*-KG, and the NAD+/NADH ratio. However, the concentrations of metabolic intermediates have not been quantified in these studies and need to be confirmed in the future. Furthermore, epigenetic alterations of certain transcripts were detected in a previous study [43]. Whether and how metabolic remodeling contributes to global changes in epigenetic events remain elusive. Modulating metabolic enzymes and nutrient availability could impact the transcriptional program of dental mesenchymal stem cell differentiation through epigenetic regulation. These metabolite-sensitive epigenetic events in differentiation processes will expand our regenerative strategies in clinical practice, such as vital pulp therapy and periodontal regeneration.

### 3.2. Aging

A long lifespan and persistence of adult stem cells contribute to a reduction in regenerative capacity [118]. The reprogramming and self-renewal ability of dental mesenchymal stem cells peaks in immature teeth and then declines with age in older teeth [91]. DPSC senescence and aging also involve metabolic remodeling characterized by a reduction in glucose, fatty acid utilization, and one-carbon metabolism-related amino acids (glycine, serine, and methionine) [59, 91]. The metabolic pathway alterations can impact the intracellular concentrations of SAM, acetyl-CoA, *α*-KG, and NAD+ and subsequently change the epigenomic states. Alterations in serine metabolism were reported to be responsible for decreased methyl donor (SAM) and enzyme activity of DNMT1 [59]. The potential role of other metabolic pathways and corresponding metabolites in epigenetic events still needs to be examined. Considering the role of metabolic signaling in senescence and aging, nutrient reservoirs can be taken as a therapeutic intervention to improve the regenerative potential of adult stem cells.

### 3.3. Inflammation

Periodontitis and pulpitis are chronic inflammatory diseases mainly caused by bacterial invasion that induce local inflammatory and immune responses. The presence of pathogens and inflammatory factors leads to metabolic remodeling that is capable of impacting the epigenetic landscape of dental mesenchymal stem cells. SCFAs derived from periodontal microbiota contribute to the intracellular acetyl-CoA generation and enzyme activity of HDACs. SCFAs play an important role in murine incisor development by facilitating the acetylation of histone H3 [94]. Meanwhile, histone acetylation induced by sodium butyrate promotes bone absorption and impairs PDLSC differentiation [110]. SCFAs are also a source of crotonyl-CoA for histone crotonylation that subsequently induces the cytokine secretion by activated macrophages [119]. These studies suggest an important role of the metabolic-epigenetic network in inflammatory disease, which provides new insight into the therapeutic strategy development in periodontitis and pulpitis.

## 4. Future Perspectives

Accumulating evidence has made it clear that the coordination of metabolic pathways and epigenetic events is essential for the transcriptomic program in cell fate decision. Metabolite-sensitive epigenetic events are highly specific, depending on different cell types and stages. Integrating metabolic cues into epigenetic events and transcriptomic programs will advance our knowledge of epitranscriptional mechanisms and provide new insight into therapeutic approaches in the oral and maxillofacial regions. Taking advantage of metabolic signaling in epigenetic regulation might help us harness a greater regenerative capacity of dental mesenchymal stem cells in vital pulp therapy and endodontic regeneration. For infectious diseases such as pulpitis and periodontitis, therapeutic inventions targeting metabolite-sensitive epigenetic events might drive inflammatory responses toward a preferable shift to injury repair. Some researchers have begun to explore metabolic remodeling in the cell fate transition of dental mesenchymal stem cells. To date, direct evidence of the metabolism-dependent epigenetic regulation of dental mesenchymal stem cells is somehow limited, and more research is needed to elucidate the exact mechanisms in the future. The metabolism-epigenetic network in dental mesenchymal cell fate specification sheds light on the therapeutic opportunity to manipulate nutrient availability and metabolite substrates and subsequently drive dental mesenchymal stem cell fate determination by epigenetic regulation.
